# An analysis of factors associated with influenza, pneumoccocal, Tdap, and herpes zoster vaccine uptake in the US adult population and corresponding inter-state variability

**DOI:** 10.1080/21645515.2017.1403697

**Published:** 2017-12-15

**Authors:** Elizabeth M. La, Laurel Trantham, Samantha K. Kurosky, Dawn Odom, Emmanuel Aris, Cosmina Hogea

**Affiliations:** a Department of Health Economics, RTI Health Solutions, Research Triangle Park, NC, USA; b Department of Biostatistics, RTI Health Solutions, Research Triangle Park, NC, USA; c Department of Real World Data and Analytics, GSK, Wavre, Belgium; d Department of Health Outcomes, GSK, Philadelphia, PA, USA

**Keywords:** adult, compliance, coverage, Influenza, herpes zoster, pneumococcal, state-level, Tdap, United States, vaccination

## Abstract

Despite longstanding recommendations for routine vaccination against influenza; pneumococcal; tetanus, diphtheria, acellular pertussis (Tdap); and herpes zoster (HZ) among the United States general adult population, vaccine uptake remains low. Understanding factors that influence adult vaccination and coverage variability beyond the national level are important steps toward developing targeted strategies for increasing vaccination coverage. A retrospective analysis was conducted using data from the Behavioral Risk Factor Surveillance System (2011–2014). Multivariable logistic regression modeling was employed to identify individual factors associated with vaccination (socio-demographics, health status, healthcare utilization, state of residence) and generate adjusted vaccination coverage and compliance estimates nationally and by state. Results indicated that multiple characteristics were consistently associated with a higher likelihood of vaccination across all four vaccines, including female sex, increased educational attainment, and annual household income. Model-adjusted vaccination coverage estimates varied widely by state, with inter-state variability for the most recent year of data as follows: influenza (aged ≥18 years) 30.2–49.5%; pneumococcal (aged ≥65 years) 64.0–74.7%; Tdap (aged ≥18 years) 18.7–46.6%; and HZ (aged ≥60 years) 21.3–42.9%. Model-adjusted compliance with age-appropriate recommendations across vaccines was low and also varied by state: influenza+Tdap (aged 18–59 years) 7.9–24.7%; influenza+Tdap+HZ (aged 60–64 years) 4.1–14.4%; and influenza+Tdap+HZ+pneumococcal (aged ≥65 years) 3.0–18.3%. In summary, after adjusting for individual characteristics associated with vaccination, substantial heterogeneity across states remained, suggesting that other local factors (e.g. state policies) may be impacting adult vaccines uptake. Further research is needed to understand such factors, focusing on differences between states with high versus low vaccination coverage.

## Highlights

• Despite recommendations for routine vaccination against influenza, pneumoccocal, Tdap, and herpes zoster in US adults, uptake remains low.
• Our study examined individual factors associated with vaccination (i.e., socio-demographics, health status, healthcare utilization, state of residence) and generated adjusted vaccination coverage/compliance estimates nationally and by state.
• Multiple characteristics were consistently associated with a higher likelihood of vaccination across all four vaccines (e.g., female sex, increased educational attainment, and annual household income).
• After adjusting for these characteristics, vaccination coverage estimates varied widely by state, suggesting that other local factors (e.g., state policies) may be impacting adult vaccination uptake.

## Introduction

The United States (US) Department of Health and Human Services estimate that approximately 42,000 adults die each year in the US from vaccine-preventable diseases.^1^ Despite efforts to reduce this burden, mortality and morbidity associated with these diseases remain high. For example, ‘influenza and pneumonia’ was the eighth leading cause of death in 2014, accounting for 2.1% of total deaths among the general population and 2.3% of total deaths among adults aged ≥65 years.^2^ The healthcare burden associated with vaccine-preventable diseases is also substantial; during the 2015-16 influenza season, the incidence of influenza-associated hospitalizations among adults aged ≥65 years was 84.8 per 100,000 population.^3^

The Advisory Committee on Immunization Practices (ACIP) currently recommends that adults in the US receive an annual seasonal influenza vaccination; an initial dose of tetanus, diphtheria, and acellular pertussis (Tdap) vaccine, with a tetanus-diphtheria (Td) booster every 10 years; a herpes zoster (HZ) vaccination for individuals aged ≥60 years; and a dose of pneumococcal 13-valent conjugate (PCV13) vaccine and pneumococcal polysaccharide (PPSV23) vaccine administered in series for individuals aged ≥65 years. Despite these long-standing guidelines, national adult vaccination coverage for routine vaccines remains low.

National adult vaccination coverage estimates based on US survey data from 2014 revealed that only 43.2% of adults aged ≥19 years had received an influenza vaccine in the 2013–14 season, 20.1% of adults aged ≥19 years had received a Tdap vaccine in the past 9 years, 27.9% of adults aged ≥60 years had ever received a HZ vaccine, and 61.3% of adults aged 65 years and older had received both pneumococcal vaccines.^4^

However, aggregating estimates of adult vaccination coverage at the national level masks state-level heterogeneity, and published state-level estimates do not typically assess compliance with age-appropriate recommendations or control for differences in health behaviors and socio-demographic factors that may be associated with vaccination uptake.^2,3,5^ Although a number of previous assessments of national vaccination surveillance data have reported heterogeneity in vaccination coverage by various patient-level characteristics,^6–9^ similar assessments at the state-level are still needed. Understanding factors that influence adult vaccination and how this impacts state-level vaccination coverage are critical steps toward developing more targeted strategies for increasing vaccination coverage.

This study investigated the association between multiple socio-demographic characteristics and health behaviors and adult influenza, pneumococcal, Tdap, and HZ vaccination. These characteristics and behaviors were further used to adjust the state-level estimates of vaccination coverage and compliance with age-appropriate recommendations. To our knowledge, no previous studies have evaluated factors associated with vaccine compliance for all four recommended vaccines in the US general adult population or generated recent national and state-level model-adjusted estimates of vaccination coverage and compliance.

## Results

The number of unweighted survey responses in each year of the Behavioral Risk Factor Surveillance System (BRFSS) data, among individuals with information on any of the vaccines of interest in the given year, ranged from nearly 465,000 in 2014 to over 506,000 in 2011; the number of weighted survey responses ranged from approximately 238.0 to 248.5 million.

In each year of the BRFSS survey, the weighted samples were generally consistent regarding demographics, health status, and health care utilization behaviors. Approximately 71-74% of the weighted sample were aged 18-59 years, 8% were aged 60-64 years, and 18-20% were aged ≥65 years. The most frequent demographic characteristics in the weighted sample included: non-Hispanic white, female, at least some education beyond high school, annual household income of at least $25,000, and health status of excellent or very good. The weighted sample sizes in each state ranged from approximately 399,000 to 426,000 in Wyoming to approximately 22 to 25 million in California.

### Vaccine coverage

Self-reported individual characteristics associated with the likelihood of influenza vaccination included female sex; increased age, level of educational attainment, and annual household income; and presence of at least one chronic condition (Supplemental Table 1). Better health status, increased time since last health check-up, and difficulties accessing care were associated with a lower likelihood of influenza vaccination. Similar relationships were generally found for pneumococcal, Tdap, and HZ vaccinations. However, the likelihood of Tdap vaccination decreased with age, and better health status was positively correlated with the likelihood of Tdap and HZ vaccination.

**Figure 1.**
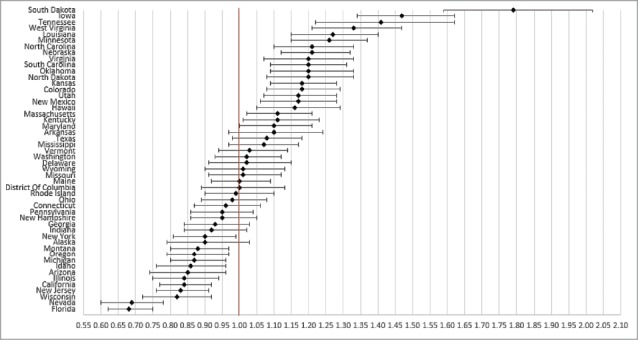
Likelihood of receipt of influenza vaccine in 2011 by state of residence versus reference state. Shown as adjusted odds ratio and 95% confidence intervals. Reference state: Alabama (alphabetic selection).

**Figure 2.**
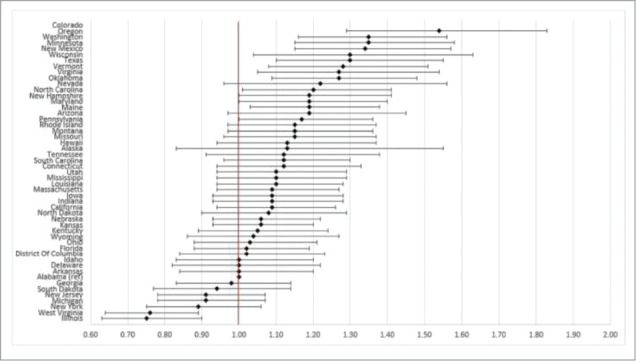
Likelihood of receipt of pneumococcal vaccine in 2011 by state of residence versus reference state. Shown as adjusted odds ratio and 95% confidence intervals. Reference state: Alabama (alphabetic selection).

**Figure 3.**
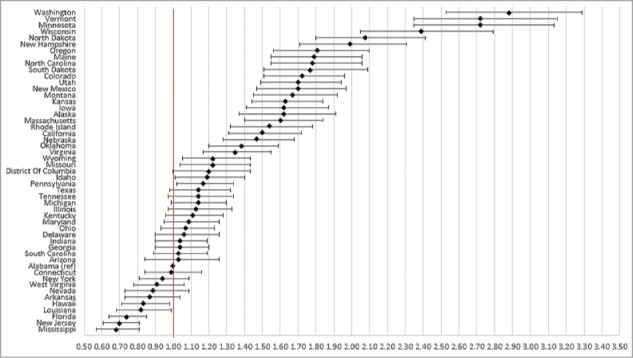
Likelihood of receipt of Tdap vaccine in 2013 by state of residence versus reference state. Shown as adjusted odds ratio and 95% confidence intervals. Reference state: Alabama (alphabetic selection).

**Figure 4.**
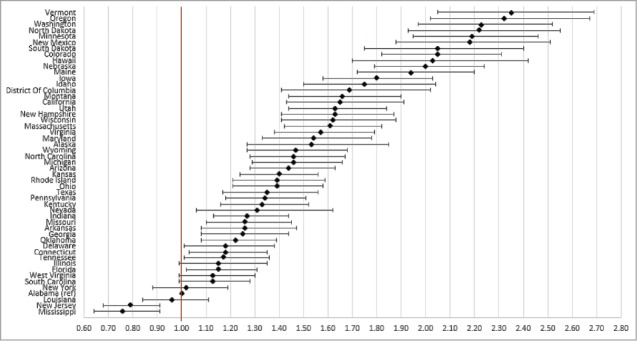
Likelihood of receipt of herpes zoster vaccine in 2014 by state of residence versus reference state. Shown as adjusted odds ratio and 95% confidence intervals. Reference state: Alabama (alphabetic selection).

State of residence also was associated with likelihood of vaccination (Figs. 1–4). Using the most recent year of data for each coverage measure, the lowest likelihood of influenza and pneumococcal vaccination was observed for individuals residing in Florida and Illinois, respectively; individuals residing in Mississippi had the lowest likelihood of both Tdap and HZ vaccination. The highest likelihood of influenza, pneumococcal, Tdap, and HZ vaccination was observed for individuals residing in South Dakota, Colorado, Washington, and Vermont, respectively.

**Figure 5.**
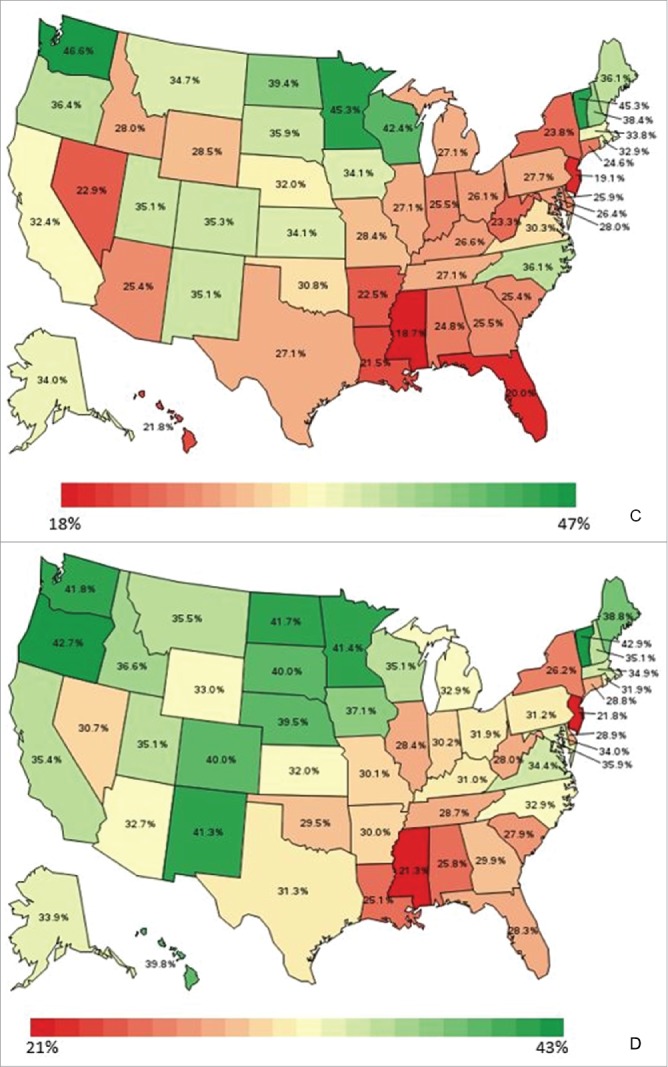
Continued.

After controlling for characteristics associated with the likelihood of vaccination, model-adjusted vaccination coverage still varied significantly by state for influenza (30.2–49.5% in 2014), pneumococcal (64.0–74.7% in 2014), Tdap (18.7–46.6% in 2013), and HZ (21.3–42.9% in 2014) (Figs. 5 A-D).
**Figure 5.**
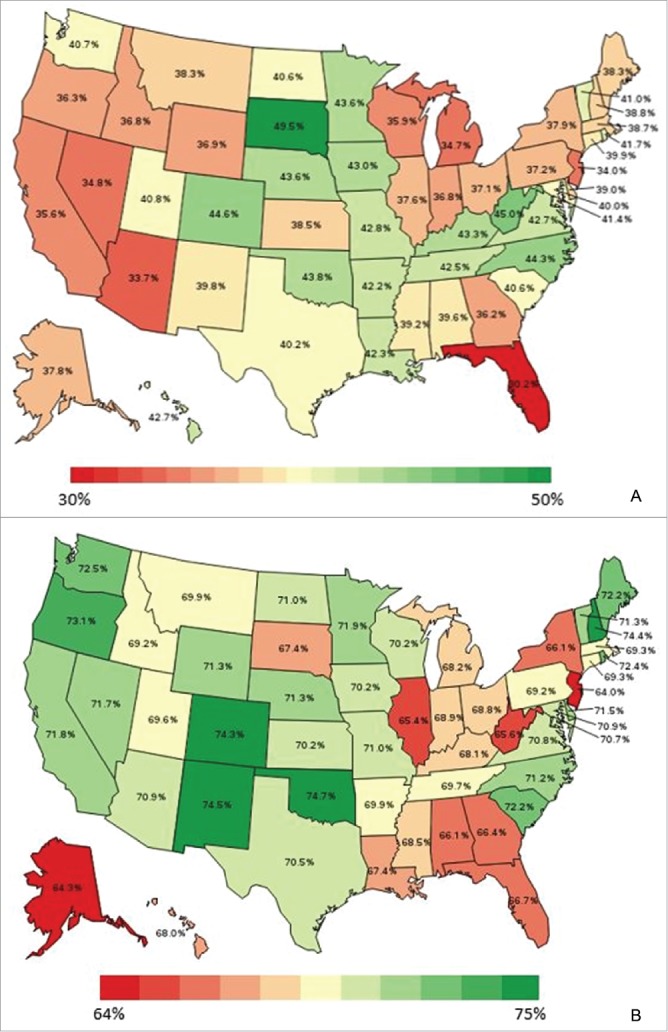
(A) Heat map of model-adjusted influenza vaccination coverage in adults aged ≥18 years in 2014. Note: Model-adjusted national influenza vaccination coverage in adults aged ≥18 years in 2014 was 38.3%. (B) Heat map of model-adjusted pneumococcal vaccination coverage in adults aged ≥65 years in 2014. Note: Model-adjusted national pneumococcal vaccination coverage in adults aged ≥65 years in 2014 was 69.4%. (C) Heat map of model-adjusted Tdap vaccination coverage in adults aged ≥18 years in 2013. Note: Model-adjusted national Tdap vaccination coverage in adults aged ≥18 years in 2013 was 28.8%. (D) Heat map of model-adjusted herpes zoster vaccination coverage in adults aged ≥60 years in 2014. Note: Model-adjusted national herpes zoster vaccination coverage in adults aged ≥60 years in 2014 was 31.8%.

Supplemental Figure 1 shows the percentile ranking of states according to the sum of z-scores for model-adjusted influenza, Tdap, pneumococcal, and HZ vaccination coverage estimates. States ranked in the bottom quartile were New Jersey, Florida, Illinois, Mississippi, New York, Nevada, Alabama, Georgia, Michigan, Connecticut, Louisiana, West Virginia, and Indiana. States ranked in the top quartile were New Hampshire, Maine, North Carolina, Nebraska, Iowa, South Dakota, North Dakota, Oregon, New Mexico, Vermont, Colorado, Washington, and Minnesota.

### Compliance with age-appropriate recommended vaccinations

#### Influenza and Tdap vaccinations among individuals aged 18–59 years

**Figure 6.**
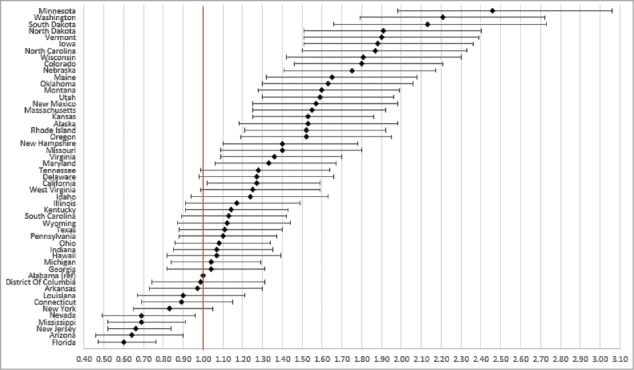
Effect of state of residence on likelihood of compliance with age-appropriate recommended influenza and Tdap vaccinations among individuals aged 18-59 years in 2013. Shown as adjusted odds ratio and 95% confidence intervals. Reference state: Alabama (alphabetic selection).

**Figure 7.**
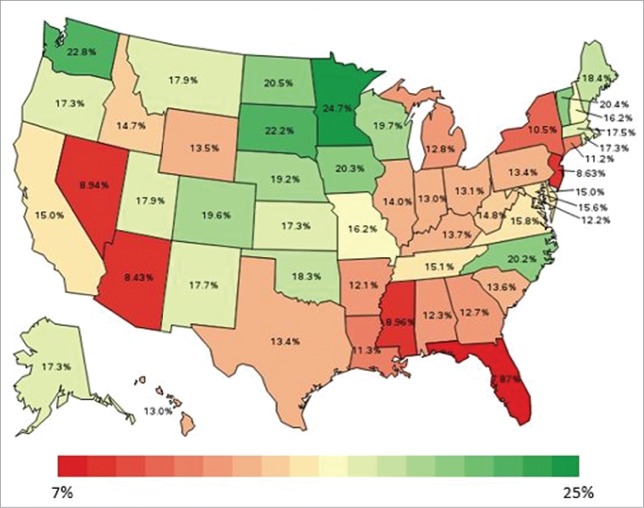
Heat map of model-adjusted compliance with age-appropriate recommended influenza and Tdap vaccinations among individuals aged 18-59 years in 2013.

Likelihood of compliance with age-appropriate influenza and Tdap vaccinations was higher among females, individuals with at least some education beyond high school, individuals with an annual household income ≥$75,000, individuals with at least one chronic condition, and individuals who did not report an inability to pay for care (Supplemental Table 2). During survey year 2013, the likelihood of compliance with age-appropriate influenza and Tdap vaccinations was lowest among individuals residing in Florida and highest among individuals residing in Minnesota (Fig. 6). After controlling for characteristics associated with the likelihood of compliance with age-appropriate recommended vaccinations among individuals aged 18-59 years, model-adjusted state-level estimates ranged from 7.9% to 24.7% in survey year 2013 (Fig. 7) and the model-adjusted national estimate was 14.3%.

#### Influenza, Tdap, and HZ vaccinations among individuals aged 60-64 years

Using data from the 10 states that included BRFSS questions related to these three vaccinations in survey year 2014, the likelihood of compliance was higher among individuals with at least some education beyond high school, individuals with an annual household income of $25,000 to <$75,000, and individuals with a very good health status (Supplemental Table 2). The likelihood of compliance with age-appropriate influenza, Tdap, and HZ vaccinations was highest for individuals residing in Vermont and lowest for individuals residing in Tennessee (Table 1). After controlling for characteristics associated with the likelihood of compliance with age-appropriate recommended vaccinations among individuals aged 60-64 years, model-adjusted state-level estimates ranged from 4.1% to 14.4% and the model-adjusted national estimate was 6.6%.

**Table 1.** Model-adjusted compliance with age-appropriate recommended vaccinations in 10 states.^a^

	Influenza, Tdap, HZ vaccines (2014)	Influenza, Tdap, HZ, pneumococcal vaccines (2014)
	Aged 60-64 years	Aged ≥65 years
	% (95% CI)	% (95% CI)
Overall compliance^b^	6.61 (5.94, 7.28)	11.21 (9.51, 12.91)
Compliance by state:		
California	5.51 (2.39, 12.17)	13.53 (9.14, 19.57)
Colorado	9.52 (6.69, 13.38)	14.82 (11.46, 18.96)
Georgia	6.12 (3.78, 9.75)	6.02 (3.81, 9.38)
Massachusetts	6.78 (4.80, 9.51)	10.32 (7.75, 13.61)
Mississippi	4.13 (1.61, 10.21)	2.99 (1.53, 5.77)
Rhode Island	5.60 (3.95, 7.89)	9.43 (6.93, 12.72)
Tennessee	4.35 (2.24, 8.27)	7.71 (5.15, 11.39)
Texas	7.76 (5.14, 11.56)	10.34 (6.89, 15.26)
Vermont	14.40 (10.74, 19.05)	18.26 (14.48, 22.76)
Virginia	7.60 (5.53, 10.36)	10.63 (7.96, 14.06)

CI, confidence interval; HZ, herpes zoster; Tdap, tetanus-diptheria-acellular pertussis

a Based on data from states which selected a BRFSS survey module that provided age-appropriate outcomes;

b Not representative at the national level

#### Influenza, Tdap, HZ, and pneumococcal vaccinations among individuals aged ≥65 years

Using data from the 10 states that included BRFSS questions related to these four vaccinations in survey year 2014, the likelihood of compliance was higher among individuals with at least some education beyond high school and among individuals aged 70–74 years, and was lower among individuals with a longer time since last check-up (Supplemental Table 2). The likelihood of age-appropriate compliance with influenza, Tdap, HZ, and pneumococcal vaccinations was highest for individuals residing in Vermont and lowest for individuals residing in Mississippi (Table 1). After controlling for characteristics associated with the likelihood of compliance with age-appropriate recommended vaccinations among individuals aged ≥65 years, model-adjusted state-level estimates ranged from 3.0% to 18.3% and the model-adjusted national estimate was 11.2%.

## Discussion

In this study we investigated the association between multiple individual-level socio-demographic, health status, healthcare utilization, and geographic factors and the receipt of influenza, pneumococcal, Tdap, and HZ vaccines among adults in the US, then generated national and state-level model-adjusted estimates of vaccination coverage and compliance with all age-appropriate recommended vaccinations. A particular focus of our analysis was on evaluating inter-state variabilities after controlling for relevant covariates. To the best of our knowledge, this is the first study that combines all of these aspects in a consistent framework across all adult vaccines currently recommended for the general US adult population.

The demographic profile of the population in our study was consistent with that of the general sampled population from the BRFSS in each of the survey years.^10–13^ In each survey year, more than half of the sample were adults aged <50 years and half were female. The majority were non-Hispanic white, had more than a high school education, and reported an annual household income of <$50,000. Most respondents reported that they had good, very good, or excellent health, although more than half of the population had at least one chronic condition; most respondents reported having a designated care provider and receiving a checkup within the past year.

Demographic characteristics such as age, sex, race, education, and income are known to be associated with receipt of adult vaccination.^14–17^ Further, health status, presence of chronic conditions, and receiving advice from health care professionals are documented factors associated with vaccine uptake.^17^ Overall, results from our multivariable regression models were generally consistent with several previous studies that have reported associations between demographic characteristics, health status, and/or health care utilization behaviors and receipt of influenza vaccination,^14–17^ Tdap vaccination,^15,18^ HZ vaccination,^9,19^ or pneumococcal vaccination.^20^

For Tdap vaccine, the likelihood of vaccination decreased with age in the current study, which may reflect the recommendations for new parents and pregnant women to receive Tdap vaccination to reduce the risk of pertussis transmission to newborns.^21–24^

Small increases in the likelihood of vaccination were observed among individuals with moderate incomes or moderate educational attainment beyond high school, although increases were greater among individuals with annual household incomes ≥$75,000 and among individuals with a college education. These findings suggest that increased vaccine coverage may be observed among individuals in the upper-middle-class demographic, who have similar levels of educational attainment and income. However, despite the findings related to income, the adjusted state-level coverage estimates observed did not necessarily align with median state-level income, suggesting that other factors may be having an influence. For example, New Jersey has a higher median income than most states, yet had vaccine coverage estimates similar to the lower median income state of Florida; New York and Wisconsin have similar median incomes,^25^ yet their vaccination coverage estimates differed substantially. These differences may be partially explained by income inequality within states, although further research is needed to investigate income inequality as a potential factor affecting vaccination coverage.

In the current study, time since last health checkup was inversely correlated with vaccination, while the presence of at least one chronic condition was associated with vaccination. Patients with chronic conditions may be a higher risk group of adults who are more likely to be targeted for vaccination by their physicians or who are better connected with their health care providers. Our study also found that individuals with difficulties accessing care were less likely to be vaccinated. Better self-reported health status was associated with a lower likelihood of influenza and pneumococcal vaccination, but a higher likelihood of Tdap and HZ vaccination. Although it is possible that individuals with worse health status have been better targeted for influenza and pneumococcal vaccinations compared with Tdap and HZ vaccinations, additional research would be needed to further investigate these associations.

In the multivariable regression analyses, even after controlling for socio-demographic, health status, and health care utilization covariates, multiple states were significantly associated with likelihood of vaccination as compared with the reference state and survey year (e.g., Alabama in 2011). The sustained effect of state of residence on likelihood of vaccination suggests that other factors such as social trends, attitudes and beliefs about vaccines, and local vaccine policies, may impact vaccination at the state level.

National model-adjusted coverage estimates were relatively low across all years and vaccines, at 36.48–38.64% for influenza vaccine coverage, 68.41–69.51% for pneumococcal vaccine coverage, 28.8% for Tdap vaccine coverage, and 31.8% for HZ vaccine coverage. However, model-adjusted vaccination coverage varied significantly by state for influenza (30.2–49.5% in 2014), pneumococcal (64.0–74.7% in 2014), Tdap (18.7–46.6% in 2013), and HZ (21.3–42.9% in 2014), highlighting substantial heterogeneity in state-level coverage.

Based on results from the ranking of states according to z-scores of model-adjusted coverage, the three lowest-ranked states were New Jersey, Florida, and Illinois. These states each had coverage estimates that were lower than the national average for all four vaccines, particularly for HZ vaccine in New Jersey, influenza vaccine in Florida, and pneumococcal vaccine in Illinois. The three highest-ranked states included Minnesota, Washington, and Colorado, which generally had coverage estimates that were higher than the national average, although these ranking results were driven by the high Tdap coverage in Minnesota and Washington, and the high pneumococcal coverage in Colorado.

The BRFSS uses a raking weighting methodology that provides a blanket adjustment for the survey's potential noncoverage and nonresponse to produce estimates that are nationally representative. However, our analyses were limited to the information reported in the BRFSS, and therefore is not an exhaustive analyses of the factors potentially associated with vaccination. For example, the analysis did not account for other factors such as vaccine-related attitudes and beliefs, local vaccine access and reimbursement, and provider and social group recommendations. Although the BRFSS data include information on whether respondents have any kind of health care coverage at the time of the survey, additional data on the type of health care coverage and whether respondents were uninsured at all in the past 12 months are only available for a subset of states. Further, information on health care coverage is not available for previous periods during which vaccines may have been received (e.g., since 2005 for the Tdap vaccine). Therefore, the current analysis relied on other data from BRFSS to measure access to care, including whether respondents reported that they needed to see a doctor in the past 12 months but could not because of cost, whether respondents reported having a designated primary care provider, and the length of time respondents reported since last doctor's visit for a routine check-up. Although these covariates are likely correlated with health care coverage status, they may provide a more direct measure of access to care since health insurance plans vary widely and having health insurance does not necessarily translate to being able to pay for and access care. Data on Tdap vaccination were obtained for the years in which Tdap vaccine questions were included in the core BRFSS survey (2013) and a BRFSS survey module that was able to be selected by states (2014). During these years, all adults were recommended to receive a Tdap vaccine, regardless of the time since last Td vaccine; however, it is possible that individuals who received a Td vaccine before the updated Tdap recommendations may have decided to wait to receive the Tdap vaccine until their booster dose was due. The BRFSS survey also does not ask about receipt of PCV13 and PPSV23 separately to determine whether individuals received the series of recommended pneumococcal vaccines.

After adjusting for individual-level covariates associated with vaccination, substantial heterogeneity across states remained in vaccination coverage and compliance with all age-appropriate recommended vaccinations. This suggests that local factors such as vaccination attitudes and beliefs, access and reimbursement, and/or local vaccination programs and policies may be having a substantial impact on adult vaccination coverage and compliance. Although these local factors were unavailable in the BRFSS data, they are an important topic for future research. For example, further research could begin to examine the correlation between state-level vaccination coverage and the types of vaccination programs and policies that are in place in each state. However, there are likely limited state-level data available on these types of local factors. Further qualitative research (e.g., interviews with key opinion leaders and policy makers within each state) may be needed to better understand the local factors influencing vaccination at the state level, particularly among states with consistently high or consistently low vaccination coverage.

## Patients and methods

### Design

This study was a self-contained, cross-sectional, retrospective exploratory database analysis of anonymized, nationally representative survey data. The aim of the study was to identify factors (e.g., socio-demographics, health status, health care utilization, and state of residence) associated with the receipt of seasonal influenza, pneumococcal, Tdap, and HZ vaccines, and subsequently generate corresponding adjusted national and state-level estimates of vaccination coverage and compliance with age-appropriate vaccine recommendations.

### Population

Data for this study were obtained from the 2011, 2012, 2013, and 2014 Behavioral Risk Factor Surveillance System (BRFSS) annual surveys.^10–13^^,^^21,26–28^ The BRFSS is an ongoing surveillance system supported by the Centers for Disease Control and Prevention (CDC) to annually monitor behavioral risk factors for non-institutionalized adults in the US using monthly random-digit-dial telephone surveys. The BRFSS uses a raking weighting methodology that provides a blanket adjustment for the survey's potential noncoverage and nonresponse to produce estimates that are representative nationally and by state (weighting includes age, sex, ethnicity, marital status, education level, home ownership, type of phone ownership, and geographic regions within states). BRFSS data prior to 2011 are not directly comparable to the more recent data being used in this study due to changes to the BRFSS weighting methodology. The BRFSS dataset includes information on the receipt of vaccines, as well as information on preventive health and risk behaviors associated with chronic diseases, injuries, and infectious diseases that may be used in an examination of individual-level factors associated with vaccination by state.

Questions related to receipt of influenza and pneumococcal vaccines were included in the standard set of questions that are asked every year by all states. Questions related to receipt of Tdap and HZ vaccines were included in the standard set of questions for 2013 and 2014, respectively; in most other years of the survey, these questions were optional and states could choose whether to include them in their questionnaires (e.g., Tdap questions were included in a module selected by 10 states in 2014).

### Outcomes

The following seven outcomes related to vaccination coverage and compliance were evaluated using available BRFSS data during the study period:

· Coverage: Receipt of a seasonal influenza vaccine in the past 12 months among individuals aged ≥18 years (2011-2014)
· Coverage: Receipt of a pneumococcal vaccine ever among individuals aged ≥65 years (2011-2014)
· Coverage: Receipt of a Tdap vaccine since 2005 among individuals aged ≥18 years (2013)
· Coverage: Receipt of a HZ vaccine ever among individuals aged ≥60 years (2014)
· Compliance: Receipt of a seasonal influenza vaccine in the past 12 months and a Tdap vaccine since 2005 among individuals aged 18–59 years (2013)
· Compliance: Receipt of a seasonal influenza vaccine in the past 12 months, a Tdap vaccine since 2005, and a HZ vaccine ever among individuals aged 60–64 years (2014, among the 10 states that asked about these 3 vaccines)
· Compliance: Receipt of a seasonal influenza vaccine in the past 12 months, a pneumococcal vaccine ever, a Tdap vaccine since 2005, and a HZ vaccine ever among individuals aged ≥65 years (2014, among the 10 states that asked about all 4 vaccines)

In the BRFSS questionnaire, respondents had the following options to answer the vaccination-related questions: yes, no, don't know/not sure, or refuse to answer. For each vaccine, crude vaccine coverage was calculated as the number of individuals who answered “yes” divided by the number of individuals who answered “yes” plus the number of individuals who answered “no”, consistent with previous analyses.^26^ To identify the socio-demographic characteristics, health status, and health care utilization behaviors associated with vaccination and compliance with age-appropriate recommended vaccination, respondents were included in each model if they answered “yes” or “no” to the relevant vaccination question(s).

The BRFSS survey asks about Td and Tdap vaccine but does not ask about history of primary Tdap vaccination. Our study uses data on Tdap vaccination from the 2013 and 2014 BRFSS surveys, when adults aged ≥18 years were recommended to receive a Tdap vaccine, regardless of the time since last Td vaccine (i.e., BRFSS respondents should have reported receiving a Tdap vaccine since 2005). Crude Tdap vaccine coverage was calculated as the number of individuals who answered that they received a Tdap vaccination since 2005 divided by the number of individuals who answered that they received a Tdap vaccination plus the number of individuals who received a tetanus vaccination that was not Tdap plus the number of individuals who did not receive a tetanus shot since 2005. Similarly, BRFSS survey questions related to pneumococcal vaccine only asked about whether the individual has ever received the pneumococcal vaccine and do not assess whether both PCV13 and PPSV23 were ever received. Therefore, coverage for pneumococcal vaccine was evaluated based on whether individuals have ever received at least one pneumococcal vaccine.

Compliance with all age-appropriate recommended vaccinations were measures of the proportions of adults within specified age ranges who received all ACIP-recommended vaccines for those age ranges (i.e., influenza/Tdap among individuals aged 18-59 years, influenza/Tdap/HZ among individuals aged 60-64 years, and influenza/Tdap/HZ/pneumococcal among individuals aged ≥65 years). The crude percentage of adults who were compliant with age-appropriate recommended vaccinations was calculated as the number of respondents who answered “yes” to receiving all age-appropriate recommended vaccinations divided by the number of respondents who answered “yes” or “no” to receiving all age-appropriate recommended vaccinations. Individuals with unknown, missing, or refused answers to any of their age-appropriate recommended vaccination questions were not included in the numerator or the denominator of the calculations.

### Covariates

Covariates were initially selected based on previous reports of an association with vaccination coverage and compliance.^7–9,15–20^ Socio-demographic covariates included age at time of BRFSS survey (18–24, 25–29, 30–34, 35–39, 40–44, 45–49, 50–54, 55–59, 60–64, 65–69, 70–74, 75–79, ≥80 years, and unknown/refused); sex; race/ethnicity (non-Hispanic white; non-Hispanic black; other race only, non-Hispanic; multiracial, non-Hispanic; Hispanic; and unknown/refused); educational attainment (less than high school, high school graduate, some college or technical school, college graduate, and unknown/refused); and annual household income (<$25,000; $25,000 to <$50,000; $50,000 to <$75,000; ≥$75,000; and unknown/refused). Health status covariates included self-reported health status and presence of at least one chronic health condition. Potential barriers to care included the inability to pay based on whether respondents reported that they needed to see a doctor in the past 12 months but could not because of cost. Health care utilization covariates included having a designated primary care provider and the length of time since last doctor's visit for a routine check-up. State of residence was also included as a covariate.

### Analyses

Multivariable logistic regression models were constructed to assess the simultaneous contribution of the covariates on each of the vaccine coverage and compliance outcomes, while accounting for the complex BRFSS survey design. All models controlled for state of residence and individual-level socio-demographic and health status characteristics and health behaviors; for models that included more than 1 year of BRFSS data, additional control variables were included for survey year and the interaction of state of residence × survey year. Including the interaction term allowed more precise state by year predictions. Fitting each multivariable model included a selection process to identify a model that balanced complexity with goodness of fit. Each covariate was individually evaluated for a significant association with the outcome through univariate testing with a pre-specified Wald test *P* value of at least 0.25 deemed significant. Next, all combinations (but not interactions) of significant measures, always including state, year, and state × year interactions, were generated systematically. Each combination was modeled for each coverage and compliance measure, with the final models selected based on the lowest Akaike Information Criterion (AIC: a commonly applied goodness of fit measure used to avoid overfitting of a model by favoring a small residual error and penalizing inclusion of extraneous covariates).^23^ For models with lower event numbers, response categories were collapsed to increase precision of the estimates).^23^ The formula used for the seven separate logistic regression models is shown in Supplement 1.^29^

Alabama was selected (alphabetically) as the referent state for regression analyses that used data from all states and the District of Columbia, and 2011 was selected as the referent survey year for regression analyses that used more than 1 year of data. Therefore, coefficients for each of the state control variables represent the difference for the given state compared with Alabama during 2011; coefficients for each of the survey year control variables were specific to the reference state; coefficients for each of the state × year interaction terms were for the given state in the given year with respect to the reference state in 2011. The regression models estimated odds ratios (ORs) and 95% confidence intervals (CIs) for each covariate, with *P* values also presented for each level of all categorical variables and for the overall significance of each categorical variable.^29^ These results were used to generate model-adjusted estimates and 95% CIs for vaccine coverage and compliance at the state and national levels by using the predicted marginal proportions from the regression models.

Model-adjusted estimates of vaccination coverage and compliance with age-appropriate recommended vaccinations are displayed graphically using heat maps of the US for the most recent year of data (for measures that included data from all states). The model-adjusted state-level coverage estimates were further used to rank states. For each vaccine-specific coverage measure in each year in which data were available nationally, z-scores were calculated for each state to provide a standardized measure of each state's deviation from the mean coverage estimate across states (with z-scores calculated as the difference between the given state's model-adjusted vaccination coverage estimate and the mean model-adjusted vaccination coverage estimate for all states, divided by the standard deviation of the model-adjusted vaccination coverage estimate for all states).^30^ For each state, these scores were summed up across vaccines in each year such that each state had a total score; years with national data available were included, with the sum of the influenza vaccine coverage scores and the sum of the pneumococcal vaccine coverage scores each weighted by ¼ to account for the 4 years of data available for these vaccines. States were ranked from lowest to highest summed z-scores, with the lowest summed z-scores corresponding to low coverage across the four vaccines and the highest summed z-scores corresponding to high coverage across the four vaccines.

All programming was conducted using SAS version 9.4 statistical software (Cary, NC: SAS Institute, Inc.; 2011) with SUDAAN 11 as a callable add-on (Research Triangle Park, NC: RTI International; 2012). RTI International's institutional review board assessed this study and determined that it did not involve identifiable human data (Federal-Wide Assurance #3331, effective until June 16, 2020).

## Supplementary Material

KHVI_A_1403697_Supplemental.zip
