# Supplementary material for: An analysis of factors associated with influenza, pneumoccocal, Tdap, and herpes zoster vaccine uptake in the US adult population and corresponding inter-state variability
Source: Hum Vaccin Immunother. 2017 Dec 15;14(2):430–41. doi: 10.1080/21645515.2017.1403697 (PMC5806688; doi:10.1080/21645515.2017.1403697)
Supplement: KHVI_A_1403697_Supplemental.zip [file khvi-14-02-1403697-s001.zip › KHVI_A_1403697_Supplemental.docx]

**Supplement 1**

Because all outcomes included in logistic regression models were dichotomous measures, the following formula was used (Bieler et al., 2010):

$logit\left( p \right)=\beta_{0}+\beta_{i}{State}_{i}+\beta_{j}{Year}_{j}+\beta_{ij}{State}_{i}{Year}_{j}+\beta_{k}X_{k},$ (Equation 1) where:

- $logit\left( p \right)$ is the log of the odds ratio (OR) that the dichotomous outcome measure of interest (i.e., coverage or compliance with age-appropriate recommended vaccinations using the outcome measures 1-7 above) is 1.
- ${State}_{i}$ is the state of residence.
- ${Year}_{j}$ is the survey year (only included for models in which more than 1 year of data is used).
- ${State}_{i}{Year}_{j}$ is the interaction term of state of residence and survey year (only included for models in which more than 1 year of data is used).
- $X_{k}$ is a vector of underlying patient characteristics (e.g., age, sex, race/ethnicity); based on the information collected in BRFSS, the 10 patient characteristics listed below were considered for inclusion in the model, contributing a maximum of approximately 43 dependent variables (including 13 variables for age, 1 variable for sex, 5 variables for race/ethnicity, 4 variables for educational attainment, 4 variables for annual household income, 5 variables for health status, 2 variables for the presence of chronic conditions, 2 variables for inability to pay for care, 2 variables for the presence of a designated care provider, and 5 variables for time since last checkup).
- β are the regression coefficients.

The estimated sign (+ or −), magnitude, and statistical significance of each β coefficient indicates the effect of the independent variable on the likelihood of the dichotomous outcome variable. In addition to raw coefficient estimates, the logistic regression models generated an OR indicating the increased or decreased likelihood of the outcome event (outcome measures 1 through 7 above). For ease of interpreting results, only the ORs are presented in the results tables. All modeling accounted for the complex BRFSS survey design.

Bieler GS, Brown GG, Williams RL, Brogan DJ. Estimating model-adjusted risks, risk differences, and risk ratios from complex survey data. Am J Epidemiol. 2010; 171(5): 618-23.

**Supplemental Table 1: Multivariable logistic regression results of characteristics associated with an individual’s likelihood of receipt of influenza, pneumococcal, Tdap, and HZ vaccines**

|  | Influenza (2011–2014) | | | Pneumococcal (2011–2014) | | | Tdap (2013) | | | HZ (2014) | | |
| --- | --- | --- | --- | --- | --- | --- | --- | --- | --- | --- | --- | --- |
|  | OR | 95% CI | *P* Value | OR | 95% CI | *P* Value | OR | 95% CI | *P* Value | OR | 95% CI | *P* Value |
| **Survey year (referent category: 2011)** | | | | | | | | | | | | |
| 2012 | **1.00** | (0.91, 1.11) | 0.9218 | **0.93** | (0.80, 1.09) | 0.3933 | ^-^ | ^-^ | ^-^ | ^-^ | ^-^ | ^-^ |
| 2013 | **1.14** | (1.02, 1.27) | 0.0197 | **0.93** | (0.78, 1.10) | 0.3936 | ^-^ | ^-^ | ^-^ | ^-^ | ^-^ | ^-^ |
| 2014 | **1.08** | (0.97, 1.19) | 0.1472 | **0.91** | (0.78, 1.07) | 0.2583 | ^-^ | ^-^ | ^-^ | ^-^ | ^-^ | ^-^ |
| **Age, years (referent category: 18-24 for influenza and Tdap; 60-64 for HZ; 65-69 for pneumococcal)** | | | | | | | | | | | | |
| 25-29 | **1.04** | (1.00, 1.08) | 0.0515 | ^-^ | ^-^ | ^-^ | **0.65** | (0.60, 0.71) | <.0001 | ^-^ | ^-^ | ^-^ |
| 30-34 | **1.07** | (1.03, 1.11) | 0.0002 | ^-^ | ^-^ | ^-^ | **0.63** | (0.58, 0.69) | <.0001 | ^-^ | ^-^ | ^-^ |
| 35-39 | **1.07** | (1.03, 1.11) | 0.0007 | ^-^ | ^-^ | ^-^ | **0.53** | (0.48, 0.58) | <.0001 | ^-^ | ^-^ | ^-^ |
| 40-44 | **1.02** | (0.98, 1.05) | 0.3879 | ^-^ | ^-^ | ^-^ | **0.42** | (0.39, 0.46) | <.0001 | ^-^ | ^-^ | ^-^ |
| 45-49 | **1.06** | (1.03, 1.10) | 0.0006 | ^-^ | ^-^ | ^-^ | **0.38** | (0.35, 0.41) | <.0001 | ^-^ | ^-^ | ^-^ |
| 50-54 | **1.20** | (1.16, 1.25) | <.0001 | ^-^ | ^-^ | ^-^ | **0.35** | (0.32, 0.38) | <.0001 | ^-^ | ^-^ | ^-^ |
| 55-59 | **1.43** | (1.39, 1.48) | <.0001 | ^-^ | ^-^ | ^-^ | **0.31** | (0.28, 0.33) | <.0001 | ^-^ | ^-^ | ^-^ |
| 60-64 | **1.74** | (1.69, 1.80) | <.0001 | ^-^ | ^-^ | ^-^ | **0.30** | (0.27, 0.32) | <.0001 | ^-^ | ^-^ | ^-^ |
| 65-69 | **2.29** | (2.21, 2.37) | <.0001 | ^-^ | ^-^ | ^-^ | **0.27** | (0.25, 0.29) | <.0001 | **1.89** | (1.78, 2.00) | <.0001 |
| 70-74 | **2.73** | (2.64, 2.83) | <.0001 | **1.65** | (1.59, 1.70) | <.0001 | **0.20** | (0.18, 0.22) | <.0001 | **2.13** | (2.00, 2.26) | <.0001 |
| 75-79 | **3.08** | (2.96, 3.20) | <.0001 | **2.13** | (2.05, 2.21) | <.0001 | **0.17** | (0.16, 0.19) | <.0001 | **2.31** | (2.16, 2.48) | <.0001 |
| ≥80 | **3.42** | (3.30, 3.55) | <.0001 | **2.43** | (2.34, 2.51) | <.0001 | **0.14** | (0.12, 0.15) | <.0001 | **2.03** | (1.90, 2.16) | <.0001 |
| **Sex (referent category: male)** | | | | | | | | | | | | |
| Female | **1.18** | (1.17, 1.20) | <.0001 | **1.26** | (1.23, 1.30) | <.0001 | **1.09** | (1.05, 1.13) | <.0001 | **1.19** | (1.14, 1.24) | <.0001 |
| **Race/ethnicity (referent category: non-Hispanic white)** | | | | | | | | | | | | |
| Non-Hispanic black | **0.75** | (0.73, 0.77) | <.0001 | **0.55** | (0.53, 0.58) | <.0001 | **0.85** | (0.80, 0.91) | <.0001 | **0.46** | (0.41, 0.52) | <.0001 |
| Other race only, Non-Hispanic | **1.16** | (1.12, 1.21) | <.0001 | **0.73** | (0.65, 0.81) | <.0001 | **0.84** | (0.75, 0.93) | 0.0012 | **0.78** | (0.65, 0.94) | 0.0081 |
| Multiracial, Non-Hispanic | **0.89** | (0.85, 0.94) | <.0001 | **0.90** | (0.80, 1.02) | 0.1009 | **0.98** | (0.84, 1.14) | 0.7757 | **0.74** | (0.60, 0.90) | 0.0025 |
| Hispanic | **1.07** | (1.04, 1.10) | <.0001 | **0.56** | (0.52, 0.60) | <.0001 | **0.90** | (0.83, 0.96) | 0.0034 | **0.60** | (0.52, 0.69) | <.0001 |
| **Educational attainment (referent category: < high school)** | | | | | | | | | | | | |
| High school graduate | **1.04** | (1.01, 1.06) | 0.0071 | **1.24** | (1.19, 1.30) | <.0001 | **1.02** | (0.94, 1.11) | 0.6015 | **1.36** | (1.24, 1.48) | <.0001 |
| Some college or technical school | **1.17** | (1.14, 1.20) | <.0001 | **1.40** | (1.33, 1.46) | <.0001 | **1.29** | (1.19, 1.40) | <.0001 | **1.55** | (1.42, 1.70) | <.0001 |
| College graduate | **1.54** | (1.50, 1.58) | <.0001 | **1.51** | (1.44, 1.59) | <.0001 | **1.58** | (1.46, 1.71) | <.0001 | **2.13** | (1.95, 2.33) | <.0001 |
| **Annual household income (referent category: < $25,000)** | | | | | | | | | | | | |
| $25,000 to < $50,000 | **1.04** | (1.02, 1.07) | <.0001 | **1.16** | (1.12, 1.20) | <.0001 | **0.95** | (0.90, 1.01) | 0.0801 | **1.28** | (1.20, 1.35) | <.0001 |
| $50,000 to < $75,000 | **1.09** | (1.06, 1.11) | <.0001 | **1.21** | (1.15, 1.27) | <.0001 | **1.05** | (0.99, 1.12) | 0.1215 | **1.63** | (1.52, 1.76) | <.0001 |
| ≥$75,000 | **1.25** | (1.22, 1.28) | <.0001 | **1.19** | (1.13, 1.24) | <.0001 | **1.14** | (1.08, 1.21) | <.0001 | **1.82** | (1.69, 1.95) | <.0001 |
| **Health status (referent category: excellent)** | | | | | | | | | | | | |
| Very good | **1.03** | (1.01, 1.05) | 0.0040 | **1.12** | (1.08, 1.17) | <.0001 | **0.96** | (0.91, 1.01) | 0.1173 | **0.96** | (0.90, 1.02) | 0.1561 |
| Good | **1.04** | (1.02, 1.07) | <.0001 | **1.30** | (1.25, 1.35) | <.0001 | **0.85** | (0.81, 0.90) | <.0001 | **0.86** | (0.81, 0.92) | <.0001 |
| Fair | **1.16** | (1.13, 1.19) | <.0001 | **1.62** | (1.54, 1.70) | <.0001 | **0.87** | (0.80, 0.93) | 0.0002 | **0.72** | (0.67, 0.78) | <.0001 |
| Poor | **1.37** | (1.32, 1.41) | <.0001 | **1.92** | (1.80, 2.05) | <.0001 | **0.81** | (0.73, 0.90) | <.0001 | **0.61** | (0.55, 0.67) | <.0001 |
| **Chronic condition (referent category: no chronic conditions)** | | | | | | | | | | | | |
| Presence of ≥1 chronic condition | **1.33** | (1.31, 1.35) | <.0001 | **1.91** | (1.85, 1.97) | <.0001 | **1.11** | (1.07, 1.16) | <.0001 | **1.26** | (1.20, 1.33) | <.0001 |
| **Inability to pay for care (referent category: needed to see doctor but could not due to cost)** | | | | | | | | | | | | |
| No inability to pay | **1.37** | (1.34, 1.41) | <.0001 | **1.09** | (1.02, 1.17) | 0.007 | **1.09** | (1.03, 1.16) | 0.0042 | **1.40** | (1.25, 1.56) | <.0001 |
| **Have designated care provider (referent category: yes)** | | | | | | | | | | | | |
| No | **0.64** | (0.63, 0.65) | <.0001 | **0.50** | (0.48, 0.53) | <.0001 | **0.73** | (0.69, 0.77) | <.0001 | **0.67** | (0.59, 0.75) | <.0001 |
| **Time since last checkup (referent category: within past year)** | | | | | | | | | | | | |
| 1-2 years | **0.63** | (0.62, 0.64) | <.0001 | **0.65** | (0.62, 0.68) | <.0001 | **0.76** | (0.72, 0.81) | <.0001 | **0.69** | (0.64, 0.75) | <.0001 |
| 2-5 years | **0.49** | (0.47, 0.50) | <.0001 | **0.50** | (0.47, 0.54) | <.0001 | **0.55** | (0.51, 0.59) | <.0001 | **0.47** | (0.42, 0.54) | <.0001 |
| ≥5 years | **0.37** | (0.35, 0.38) | <.0001 | **0.37** | (0.35, 0.40) | <.0001 | **0.36** | (0.33, 0.39) | <.0001 | **0.31** | (0.27, 0.35) | <.0001 |
| Never | **0.47** | (0.43, 0.51) | <.0001 | **0.41** | (0.36, 0.48) | <.0001 | **0.34** | (0.28, 0.42) | <.0001 | **0.47** | (0.35, 0.64) | <.0001 |

CI, confidence interval; HZ, herpes zoster; Tdap, tetanus-diphtheria-acellular pertussis; OR: Odds ratio; CI: confidence interval

Note: The logistic regression models of the individual-level likelihood of vaccination were based on 1,793,084 observations and 823,884 events for influenza vaccine, 567,395 observations and 398,941 events for pneumococcal vaccine, 288,560 observations and 80,189 events for Tdap vaccine, and 195,626 observations and 70,157 events for HZ vaccine. The c-statistics for regression models ranged from 0.680 to 0.719.

**Supplemental Table 2: Multivariable logistic regression results of characteristics associated with the likelihood of compliance with age-appropriate recommended vaccinations**

|  | Model 1  Received Age-Appropriate Recommended Influenza and Tdap Vaccinations Among Individuals Aged 18–59 Years (2013) | | | Model 2  Received Age-Appropriate Recommended Influenza, Tdap, and HZ Vaccinations Among Individuals Aged 60–64 years (2014) | | | Model 3  Received Age-Appropriate Recommended Influenza, Pneumococcal, Tdap, and HZ Vaccinations Among Individuals Aged ≥65 years (2014) | | |
| --- | --- | --- | --- | --- | --- | --- | --- | --- | --- |
|  | OR | 95% CI | *P* Value | OR | 95% CI | *P* Value | OR | 95% CI | *P* Value |
| **State (referent category: California)** | | | | | | | | | |
| Colorado | *See Figure 6* | | | **1.88** | (0.70, 5.04) | 0.2080 | **1.13** | (0.70, 1.83) | 0.6188 |
| Georgia |  |  |  | **1.13** | (0.40, 3.16) | 0.8224 | **0.37** | (0.21, 0.64) | 0.0004 |
| Massachusetts |  |  |  | **1.27** | (0.50, 3.20) | 0.6185 | **0.71** | (0.44, 1.13) | 0.1487 |
| Mississippi |  |  |  | **0.73** | (0.19, 2.87) | 0.6518 | **0.17** | (0.08, 0.34) | < 0.0001 |
| Rhode Island |  |  |  | **1.02** | (0.40, 2.60) | 0.9677 | **0.63** | (0.39, 1.03) | 0.0636 |
| Tennessee |  |  |  | **0.77** | (0.25, 2.35) | 0.6473 | **0.50** | (0.29, 0.86) | 0.0126 |
| Texas |  |  |  | **1.48** | (0.54, 4.03) | 0.4450 | **0.71** | (0.40, 1.25) | 0.2363 |
| Vermont |  |  |  | **3.17** | (1.24, 8.11) | 0.0159 | **1.51** | (0.94, 2.40) | 0.0851 |
| Virginia |  |  |  | **1.44** | (0.57, 3.67) | 0.4418 | **0.73** | (0.45, 1.20) | 0.2177 |
| **Age, years (referent category: 18-24 for Model 1; 65-69 for Model 3)** | | | | | | | | | |
| 25-29 | **0.96** | (0.85, 1.08) | 0.4607 | ^-^ | ^-^ | ^-^ | ^-^ | ^-^ | ^-^ |
| 30-34 | **0.93** | (0.83, 1.04) | 0.2084 | ^-^ | ^-^ | ^-^ | ^-^ | ^-^ | ^-^ |
| 35-39 | **0.81** | (0.72, 0.92) | 0.0012 | ^-^ | ^-^ | ^-^ | ^-^ | ^-^ | ^-^ |
| 40-44 | **0.65** | (0.58, 0.73) | < 0.0001 | ^-^ | ^-^ | ^-^ | ^-^ | ^-^ | ^-^ |
| 45-49 | **0.60** | (0.53, 0.68) | < 0.0001 | ^-^ | ^-^ | ^-^ | ^-^ | ^-^ | ^-^ |
| 50-54 | **0.64** | (0.57, 0.73) | < 0.0001 | ^-^ | ^-^ | ^-^ | ^-^ | ^-^ | ^-^ |
| 55-59 | **0.62** | (0.55, 0.69) | < 0.0001 | ^-^ | ^-^ | ^-^ | ^-^ | ^-^ | ^-^ |
| 60-64 | **^-^** | ^-^ | ^-^ | ^-^ | ^-^ | ^-^ | ^-^ | ^-^ | ^-^ |
| 65-69 | **^-^** | ^-^ | ^-^ | ^-^ | ^-^ | ^-^ | ^-^ | ^-^ | ^-^ |
| 70-74 | **^-^** | ^-^ | ^-^ | ^-^ | ^-^ | ^-^ | **1.89** | (1.10, 3.26) | 0.0222 |
| 75-79 | **^-^** | ^-^ | ^-^ | ^-^ | ^-^ | ^-^ | **0.92** | (0.47, 1.78) | 0.8002 |
| ≥80 | **^-^** | ^-^ | ^-^ | ^-^ | ^-^ | ^-^ | **0.76** | (0.35, 1.65) | 0.4927 |
| **Sex (referent category: male)** | | | | | | | | | |
| Female | **1.24** | (1.16, 1.31) | < 0.0001 | ^-^ | ^-^ | ^-^ | **0.92** | (0.59, 1.44) | 0.7247 |
| **Race/ethnicity (referent category: non-Hispanic white)** | | | | | | | | | |
| Non-Hispanic black | **0.74** | (0.67, 0.83) | < 0.0001 | ^-^ | ^-^ | ^-^ | **0.80** | (0.24, 2.62) | 0.7075 |
| Other race only, Non-Hispanic | **0.98** | (0.84, 1.14) | 0.7856 | ^-^ | ^-^ | ^-^ | **2.64** | (0.94, 7.37) | 0.0644 |
| Multiracial, Non-Hispanic | **0.87** | (0.72, 1.07) | 0.1883 | ^-^ | ^-^ | ^-^ | **1.31** | (0.37, 4.70) | 0.6760 |
| Hispanic | **0.96** | (0.86, 1.07) | 0.4278 | ^-^ | ^-^ | ^-^ | **2.32** | (0.92, 5.84) | 0.0744 |
| Other race | **^-^** | ^-^ | ^-^ | **0.42** | (0.17, 1.06) | 0.0664 | ^-^ | ^-^ | ^-^ |
| **Educational attainment (referent category: < high school for Models 1 and 3; ≤ high school graduate for Model 2)** | | | | | | | | | |
| High school graduate | **1.05** | (0.90, 1.22) | 0.5387 | ^-^ | ^-^ | ^-^ | **1.66** | (0.67, 4.15) | 0.2759 |
| Some college or technical school | **1.37** | (1.19, 1.58) | < 0.0001 | **5.97** | (2.68, 13.30) | < 0.0001 | **2.39** | (1.14, 4.99) | 0.0207 |
| College graduate | **1.87** | (1.62, 2.17) | < 0.0001 | **7.26** | (3.45, 15.30) | < 0.0001 | **3.29** | (1.55, 6.99) | 0.0019 |
| **Annual household income (referent category: < $25,000)** | | | | | | | | | |
| $25,000 to < $50,000 | **0.97** | (0.88, 1.08) | 0.6138 | **2.66** | (1.04, 6.81) | 0.0412 | **0.90** | (0.46, 1.76) | 0.7655 |
| $50,000 to < $75,000 | **1.08** | (0.97, 1.20) | 0.1434 | **2.93** | (1.09, 7.87) | 0.0326 | **0.96** | (0.38, 2.40) | 0.9293 |
| ≥$75,000 | **1.25** | (1.14, 1.37) | < 0.0001 | **2.19** | (0.98, 4.90) | 0.0562 | **1.24** | (0.58, 2.66) | 0.5778 |
| **Health status (referent category: excellent)** | | | | | | | | | |
| Very good | **0.91** | (0.85, 0.98) | 0.0123 | **2.06** | (1.15, 3.69) | 0.0150 | **0.74** | (0.40, 1.34) | 0.3194 |
| Good | **0.81** | (0.75, 0.88) | < 0.0001 | **1.16** | (0.49, 2.74) | 0.7280 | **0.39** | (0.20, 0.76) | 0.0054 |
| Fair | **0.92** | (0.81, 1.05) | 0.2107 | **1.62** | (0.59, 4.48) | 0.3535 | **0.51** | (0.22, 1.15) | 0.1034 |
| Poor | **0.92** | (0.77, 1.09) | 0.3199 | **0.37** | (0.08, 1.73) | 0.2062 | **0.23** | (0.09, 0.62) | 0.0035 |
| **Chronic condition (referent category: no chronic conditions)** | | | | | | | | | |
| Presence of ≥1 chronic condition | **1.16** | (1.10, 1.23) | < 0.0001 | ^-^ | ^-^ | ^-^ | **0.96** | (0.59, 1.55) | 0.8694 |
| **Inability to pay for care (referent category: needed to see doctor but could not due to cost)** | | | | | | | | | |
| No inability to pay | **1.39** | (1.27, 1.52) | < 0.0001 | **1.53** | (0.62, 3.79) | 0.3602 | **1.85** | (0.76, 4.50) | 0.1783 |
| **Have designated care provider (referent category: yes)** | | | | | | | | | |
| No | **0.59** | (0.54, 0.64) | < 0.0001 | ^-^ | ^-^ | ^-^ | ^-^ | ^-^ | ^-^ |
| **Time since last checkup (referent category: within past year)** | | | | | | | | | |
| 1-2 years | **0.63** | (0.58, 0.69) | < 0.0001 | ^-^ | ^-^ | ^-^ | **0.33** | (0.15, 0.72) | 0.0057 |
| 2-5 years | **0.47** | (0.42, 0.54) | < 0.0001 | ^-^ | ^-^ | ^-^ | ^-^ | ^-^ | ^-^ |
| ≥5 years | **0.28** | (0.24, 0.32) | < 0.0001 | ^-^ | ^-^ | ^-^ | ^-^ | ^-^ | ^-^ |
| Never | **0.45** | (0.31, 0.64) | < 0.0001 | ^-^ | ^-^ | ^-^ | ^-^ | ^-^ | ^-^ |
| ≥2 years or never | ^-^ | ^-^ | ^-^ | ^-^ | ^-^ | ^-^ | **0.21** | (0.06, 0.75) | 0.0165 |

CI, confidence interval; HZ, herpes zoster; Tdap, tetanus-diphtheria-acellular pertussis; OR: Odds ratio; CI: confidence intervalNote: The logistic regression models of the individual-level likelihood of compliance with age-appropriate recommended vaccinations were based on 156,759 observations and 25,978 events (i.e., respondents who were compliant with the age-appropriate recommendations) for influenza and Tdap vaccines; 4,398 observations and 422 events for influenza, Tdap, and HZ vaccines; and 12,611 observations and 1,135 events for influenza, Tdap, HZ, and pneumococcal vaccines. The c-statistics for regression models ranged from 0.697 to 0.719.

**Supplemental Figure 1: Ranking of states based on the sum of z-scores for model-adjusted influenza, Tdap, pneumococcal, and herpes zoster vaccination coverage estimates**

Note: States were ranked according to the sum of each state’s calculated z-score for each vaccine, providing a composite metric across all four vaccines. Z-scores were used for each vaccine to provide a standardized measure of each state’s deviation from the mean model-adjusted coverage estimate across states (i.e., each state’s z-score for a particular vaccine represents the number of standard deviations that the state’s model-adjusted vaccine coverage estimate is from the mean model-adjusted coverage estimate).
