# Supplementary figures and images for: An analysis of factors associated with influenza, pneumoccocal, Tdap, and herpes zoster vaccine uptake in the US adult population and corresponding inter-state variability
Source: Hum Vaccin Immunother. 2017 Dec 15;14(2):430–41. doi: 10.1080/21645515.2017.1403697 (PMC5806688; doi:10.1080/21645515.2017.1403697)

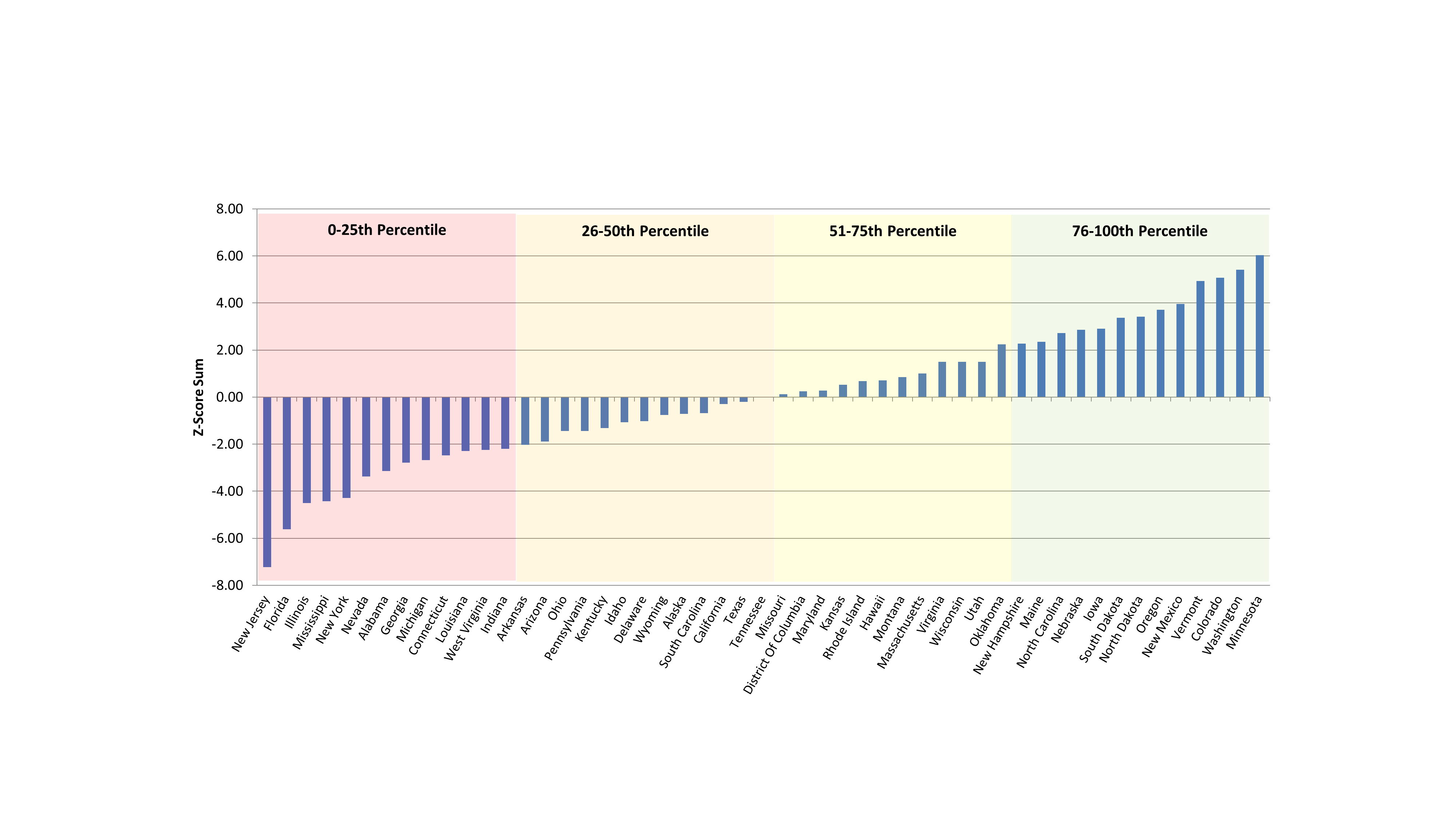

Supplement: KHVI_A_1403697_Supplemental.zip [file khvi-14-02-1403697-s001.zip › KHVI_A_1403697_Figure S1.tif]
